# Changes in the contents of four active diterpenoids at different growth stages in *Andrographis paniculata* (Burm.f.) Nees (*Chuanxinlian*)

**DOI:** 10.1186/1749-8546-8-2

**Published:** 2013-01-15

**Authors:** Nanthanit Pholphana, Nuchanart Rangkadilok, Jittra Saehun, Supachai Ritruechai, Jutamaad Satayavivad

**Affiliations:** 1 Laboratory of Pharmacology, Chulabhorn Research Institute (CRI), Kamphaeng Phet 6 Road, Laksi, Bangkok, 10210, Thailand; 2 Chulabhorn Graduate Institute (CGI), Kamphaeng Phet 6 Road, Laksi, Bangkok, 10210, Thailand; 3 Center of Excellence on Environmental Health and Toxicology, Office of the Higher Education Commission, Ministry of Education, Bangkok, 10400, Thailand

## Abstract

**Background:**

The therapeutic activities of *Andrographis paniculata* are attributed to four major active diterpenoids: andrographolide (AP_1_), 14-deoxy-11,12-didehydroandrographolide (AP_3_), neoandrographolide (AP_4_), and 14-deoxyandrographolide (AP_6_). This study aims to quantify the four active diterpenoids in various plant organs of *A. paniculata* at different growth stages in greenhouse and field experiments, with a developed HPLC-diode array detector (HPLC-DAD) method for simultaneous determination of these diterpenoids.

**Methods:**

Plants were grown in greenhouse and in field conditions, harvested at different growth stages, and separated into different organs for determination of the four active diterpenoids by an HPLC-DAD method.

**Results:**

The most abundant diterpenoid was AP_6_ between seedling and vegetative stages in the greenhouse experiment (13.38 to 23.71 mg/g in 2006 and 10.67 to 24.54 mg/g in 2007). High levels of AP_6_ were also detected in leaves at the transfer stage in the greenhouse experiment (36.05 ± 0.69 mg/g) and field experiment (30.59 ± 1.39 mg/g). The levels of AP_6_ then decreased as plants matured. The highest content of AP_4_ was in cotyledons (16.65 ± 4.48 mg/g) at the transfer stage. The highest contents of AP_1_ were detected in leaves at seed-forming stage in greenhouse experiment (24.72 ± 1.89 mg/g) and vegetative stage in field experiment (43.16 ± 0.92 mg/g). Flowers of *A. paniculata* contained high levels of AP_1_ (21.42 ± 3.74 mg/g). AP_3_ and AP_4_ were at low levels in leaves at all growth stages.

**Conclusion:**

In *A. paniculata*, AP_6_ was at the highest level in leaves at transfer stage in both greenhouse and field experiments. AP_1_ was at the highest level in leaves at vegetative stage and seed-forming stage in field and greenhouse experiments, respectively. The contents of AP_3_ and AP_4_ in leaves were low at all growth stages.

## Background

*Andrographis paniculata* (Burm.f.) Nees, (Acanthaceae) (known as *Chuanxinlian* in China) is used in Scandinavian and Asian countries to treat the common cold, fever, and diarrhea [1]. The aerial part of *A. paniculata* is used in traditional Chinese medicine [2]. *A. paniculata* is beneficial to the liver, immune system, respiratory system, and cardiovascular system, and shows anti-inflammatory, antimalarial, antidiarrheal, hypoglycemic, anti-fertility, anticancer, and anti-HIV activities [1-4]. These therapeutic activities of *A. paniculata* are attributed to four active diterpenoids; andrographolide (AP_1_), 14-deoxy-11,12-didehydroandrographolide (AP_3_), neoandrographolide (AP_4_), and 14-deoxyandrographolide (AP_6_) [1-4], which exhibit different pharmacological activities [2,4]. AP_1_ showed anti-inflammatory [5] and anticancer activities with stronger effects on diverse cancer cells than AP_3_ and AP_6_[6]. It also showed cardioprotective properties [7,8]. Our previous studies indicated that AP_3_ had a potent hypotensive effect [9] and higher anti-platelet activity than AP_1_[10]. AP_4_ scavenged free radicals [11], and showed stronger anti-inflammatory activity than AP_1_ by inhibiting nitric oxide production both *in vitro* and *ex vivo*[12]. AP_4_ also had antimalarial activities [13] and hepatoprotective effects against carbon tetrachloride [14]. AP_6_ is an effective antagonist of platelet activating factor-mediated processes in bovine neutrophils via its effects on calcium channels [15]. It also caused vasorelaxation of rat thoracic aorta [16] and relaxation of uterine smooth muscle via selectively blocking voltage-operated calcium channels [17].

There is a large market requirement for *A. paniculata* materials, with an estimated consumption in India (aerial part) of 250 tonnes per year [18]. *A. paniculata* is now commercially cultivated to meet the high demand [19]. There have been several studies on cultivation of *A. paniculata*. Kumar *et al.*[20] reported that 25°C was the optimal germination temperature (94.6% germination), while there was no germination at 40°C. The growth regulators abscisic acid and gibberellic acid also increased the AP_1_ content [21]. There are few reports on the contents of active diterpenoids in *A. paniculata* during plant development. Bhan *et al.*[22] analyzed the contents of three diterpenoids in leaves at three harvesting dates. Prathanturarug *et al.*[23] investigated the contents of AP_1_ and AP_3_ in field-grown *A. paniculata* harvested at the 60% flowering stage. Parasher *et al.*[24] reported that the maximum AP_1_ content in leaves was at 120 days of maturity. There were large variations in the contents of three active diterpenoids (AP_1_, AP_3_, and AP_4_) among different *A. paniculata* products in a Thai market [25]. Both the growing region and season strongly affect production of the diterpene lactones in this plant [1].

In this study, we developed a simple and rapid extraction method followed by simultaneous determination of the four active diterpenoids (AP_1_, AP_3_, AP_4_, and AP_6_) in different parts of *A. paniculata.* This simple reversed-phase HPLC-diode array detector (DAD) method was successfully applied to quantify the major components in *A. paniculata* at different growth stages*.* The method is sufficiently rapid, simple, sensitive, and cost-effective to be used for quality control of raw materials and herbal preparations [25]. DAD can be used to identify the active compounds based on their absorption spectra.

To our knowledge, there are no reports on the contents of the four active diterpenoids at different growth stages from seedling to mature stages of *A. paniculata*. In addition, there are few reports on the contents of these four active compounds in various plant organs at different growth stages. The aim of this study was to determine the changes in contents of the four active diterpenoids in various plant organs of *A. paniculata* at different growth stages in greenhouse and field experiments at two different cultivation times: September 2006 and January 2007.

## Methods

### Chemicals

**Figure 1:**
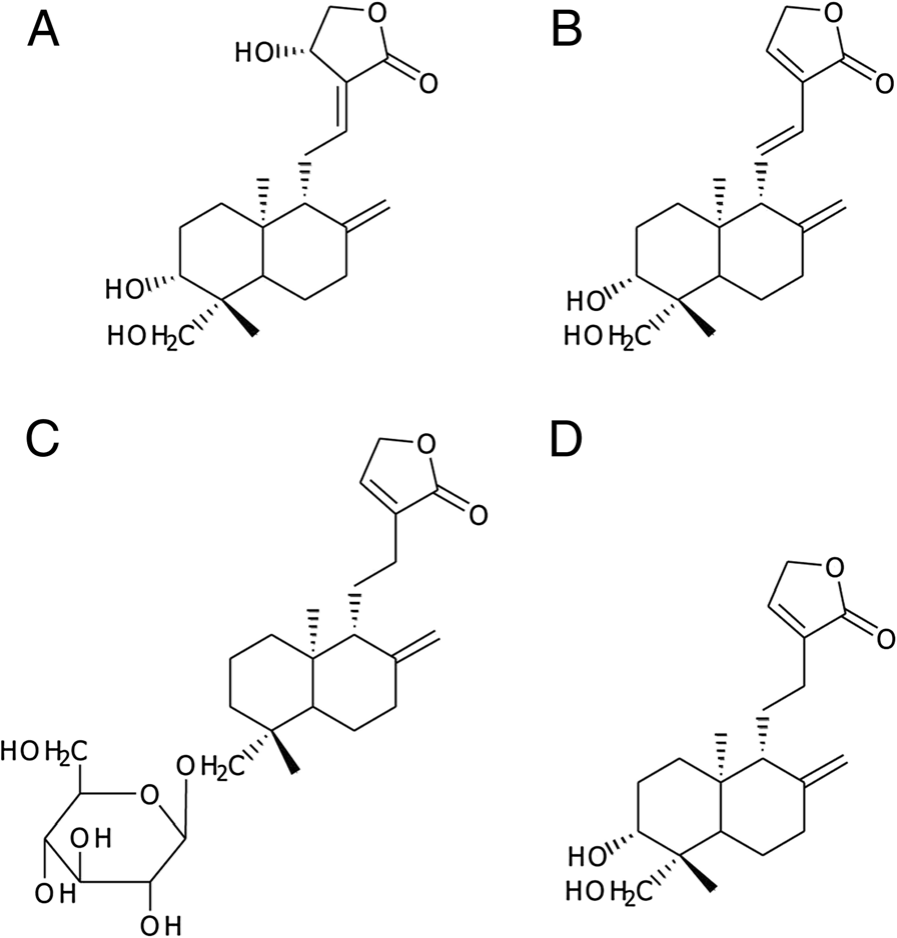
**Structures of four main active diterpenoids in *Andrographis paniculata. *A**. andrographolide (AP_1_); **B**. 14-deoxy-11,12-didehydroandrographolide (AP_3_); **C**. neoandrographolide (AP_4_); and **D**. 14-deoxyandrographolide (AP_6_).

Four standard diterpenoids, andrographolide (AP_1_, purity 99%), 14-deoxy-11,12-didehydroandrographolide (AP_3_, purity 96%), neoandrographolide (AP_4_, purity 99%), and 14-deoxyandrographolide (AP_6_, purity 99%) were purified at our Institute by the Laboratory of Pharmacology and Laboratory of Natural Products, Chulabhorn Research Institute (CRI), Thailand, following a method published previously [26]. They were extracted from *A. paniculata* plant materials and identified by thin-layer chromatography (TLC), ultraviolet (UV) spectra, melting point (MP) analysis, infrared (IR) analysis, mass spectrometry (MS), and nuclear magnetic resonance (NMR) spectroscopy. The structures of these four active diterpenoids are shown in Figure 1. HPLC grade methanol and acetonitrile were obtained from Merck (Darmstadt, FR, Germany). High purity water obtained from a Milli-Q water purification system (Millipore, Bedford, MA, USA) was used in these experiments.

### Plant materials

#### Greenhouse experiment

*A. paniculata* plants were identified by Dr. Wongsatit Chuakul and a voucher specimen was deposited at the Pharmaceutical Botany Mahidol Herbarium, Department of Pharmaceutical Botany, Faculty of Pharmacy, Mahidol University, Bangkok, Thailand (PBM 3760). In the greenhouse experiment, we studied two cultivation times: September 2006 and January 2007. The temperature ranged from 24.2 to 28.5°C and the relative humidity was from 60 to 70% in the greenhouse environment. Seeds were sown in a tray and grown in the greenhouse at CRI. Peat moss soil (Chia Tai, Bangkok, Thailand) was used in the greenhouse experiments (pH 5.5; N-P-K 12-14-24; 0.8 kg/m^3^). At the seedling, first true leaf, and transfer stages, plants were grown in the tray. At the transfer stage, plants were transplanted into pots to grow on to the mature stages (vegetative, 50% flowering, seed-forming, and mature-seed stages). Plants were harvested at different growth stages and separated into different parts (cotyledon, hypocotyl, leaf, stem, flower, young pod including seed, pod, root, and mature seed) depending on the growth stage (Table 1) to quantify the four active diterpenoids. Plant materials were washed and then dried in an oven at 35–45°C [27]. The dried plant materials were ground into a powder using a blender (Waring Commercial, CT, USA), and kept at room temperature until extraction and analysis.

**Table 1:** ***Andrographis paniculata *materials from plants at various developmental stages in greenhouse experiment in two growing seasons, September 2006, and January 2007**

**Stages**	**Harvesting time (days)**		**Plant organs**
	**September 2006**	**January 2007**	
Tray			
1. Seedling	7	7	Cotyledon, hypocotyl, root
2. First true leaf	14	14	Cotyledon, young leaf, stem, root
3. Transfer	39	45	Cotyledon, leaf, stem, root
Pot			
4. Vegetative	108	114	Leaf, stem, root
5. 50% Flowering	128	141	Leaf, stem, root, flower, young pod (including seed)
6. Seed forming	133	149	Leaf, stem, root, flower, young pod (including seed)
7. Mature seed	161	206	Leaf, stem, root, pod, and seed

#### Field experiment

Seeds were sown in the field at Ratchaburi Province, Thailand. Plants were harvested at different growth stages; transfer, vegetative, 50% flowering, seed-forming, and mature-seed stages (2, 3.5, 5, 6, and 6.5 months old, respectively) to quantify the four active diterpenoids. Sun-dried plant materials from the field were separated into two parts (leaves and aerial part). The dried leaves and aerial parts were ground into powder, and kept at room temperature until extraction and analysis.

### Sample preparation

#### Greenhouse experiment

Dried powder (0.1 g) of *A. paniculata* from different plant organs at different growth stages was accurately weighed (three replicates per sample, 20 plants per replicate at seedling, first true leaf, and transfer stages, two plants per replicate at other maturity stages). The plant powder was extracted with 4 mL methanol and rotated with a rugged rotator (Glas-Col, IN, USA) for 5 min, and then centrifuged (Sorvall, Munich, Germany) at 1876 *× g* for 10 min. The supernatant was transferred into a 10 mL volumetric flask and the crude powder was re-extracted twice with 3 mL methanol. All extracts were combined, the volume was adjusted, and then the solution was filtered through a 0.45-μm nylon membrane (Chrom Tech, MN, USA) before HPLC analysis. The extraction method was modified from our previous study [25].

#### Field experiment

Powdered plant material (leaves or aerial part) (0.1 g) of *A. paniculata* at different growth stages was accurately weighed (three replicates per sample, 20 plants per replicate at transfer stage, two plants per replicate at other maturity stages) and then extracted as described above for the greenhouse experiment.

### HPLC-DAD

The four compounds in the methanolic extract of *A. paniculata* were analyzed simultaneously by HPLC-DAD (Agilent Technologies, Germany) on a reverse phase column (Zorbax SB-C18; 4.6 × 75 mm, 3.5 μm) connected to a cartridge guard column (Agilent Technologies, USA). The simple mobile phase consisted of 28% acetonitrile in water with a flow rate of 1.2 mL/min. The temperature of the column was controlled at 25°C and the detection with the diode array detector (DAD; Model G1315A, Agilent Technologies, Germany) at 205 nm. The injection volume was 5 μL. A standard mixture containing AP_1_, AP_3_, AP_4_, and AP_6_ in methanol was prepared in the range of 0.5–1000 μg/mL. The peak area of each compound was plotted against the concentration.

### Statistical analysis

Data are expressed as mean ± standard deviation (SD, *n* = 3). Linear regression analysis was used to compare DAD responses and concentrations using ChemStation software version A.10.02 (Agilent Technologies, Germany). Accuracy was accessed as percentage recovery using the following formula: $\left(\frac{\left(C_{\text{Spiked}}-C_{\text{Sample}}\right)}{C_{\text{Standard}\;\text{added}}}\times 100\right)$. Precision was expressed as the percent relative standard deviation; %RSD $\left(\text{\%RSD}=\frac{\text{SD}}{\overset{―}{X}}\times 100\right)$, which was calculated with Excel 2010 software (Microsoft, USA).

## Results and discussion

### Method validation

**Figure 2:**
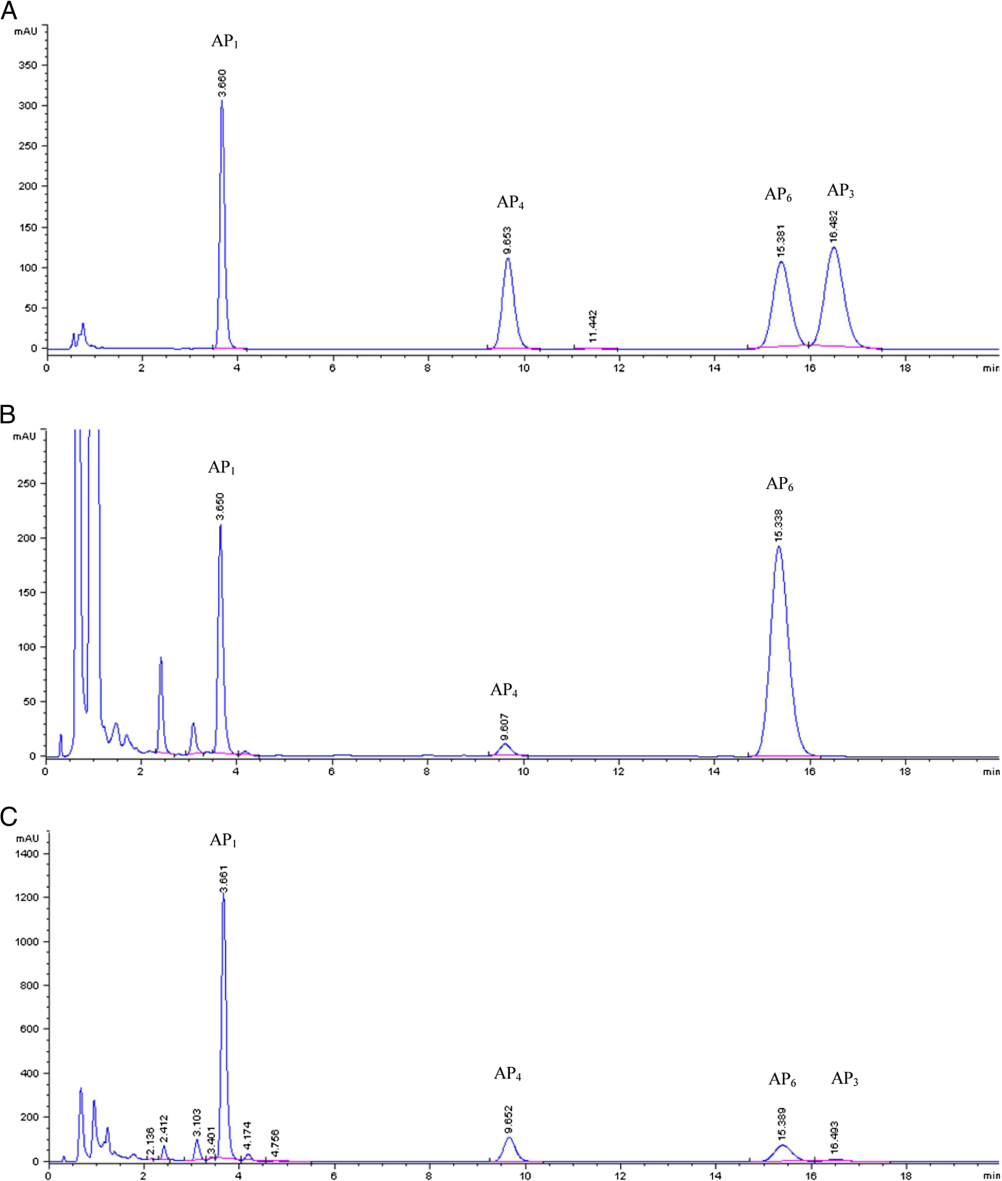
**HPLC chromatograms of four diterpenoids in *Andrographis paniculata. *A**. Four standard diterpenoids; **B**. Leaf sample of *Andrographis paniculata* at transfer stage; and **C**. Leaf sample of *Andrographis paniculata* at mature-seed stage.

Xu *et al.*[28] determined diterpenoids in *A. paniculata* leaves by silver ion complexation in the mobile phase to separate AP_3_ and AP_6_ with a 25-min analysis time. The analysis time in the present study was less than 18 min. The chromatograms in Figure 2 showed good separation of these four active diterpenoids; a chromatogram of the standard solution is shown, compared with samples containing the highest contents of AP_6_ and AP_1_ at the transfer and mature-seed stages, respectively. The calibration curves showed good linearity in the range of 0.5 to 1000 μg/mL, and the correlation coefficients were 0.9999 for AP_1_, AP_3_, AP_4_, and AP_6_. Accuracy was determined (expressed as percentage recovery) by adding three known amounts of standard solutions to the plant samples, 10 replicates per concentration, and then extracting the samples and analyzing by the HPLC method described above. The mean recoveries of the four active diterpenoids, AP_1_, AP_3_, AP_4_, and AP_6_, were 99.66 ± 2.22%, 98.91 ± 2.29%, 100.20 ± 1.85%, and 97.98 ± 1.90%, respectively, indicating good accuracy of the method. To access the precision of the method, we analyzed the extracted sample solutions 10 times on the same day and 10 times on 3 consecutive days. The results showed acceptable intra- and inter-day precision, as represented by the percent relative standard deviation (%RSD) ranging from 0.80% to 1.02% (content) and from 0.41% to 0.66% (retention time) for intra-day precision, and from 3.42% to 4.92% (content) and from 0.39% to 0.74% (retention time) for inter-day precision. The limit of detection (LOD) and limit of quantitation (LOQ) were measured based on the signal to noise ratio of 3:1 and 10:1, respectively. The LOD of AP_1_, AP_3_, AP_4_, and AP_6_ were 0.10, 0.25, 0.25, and 0.25 μg/mL, respectively, and the LOQ were 0.25, 0.5, 0.5, and 0.5 μg/mL, respectively. These results showed that our analytical method met acceptable criteria for all analytes, and could be used for routine analysis for the four active diterpenoids in *A. paniculata*.

### Greenhouse experiment

**Figure 3:**
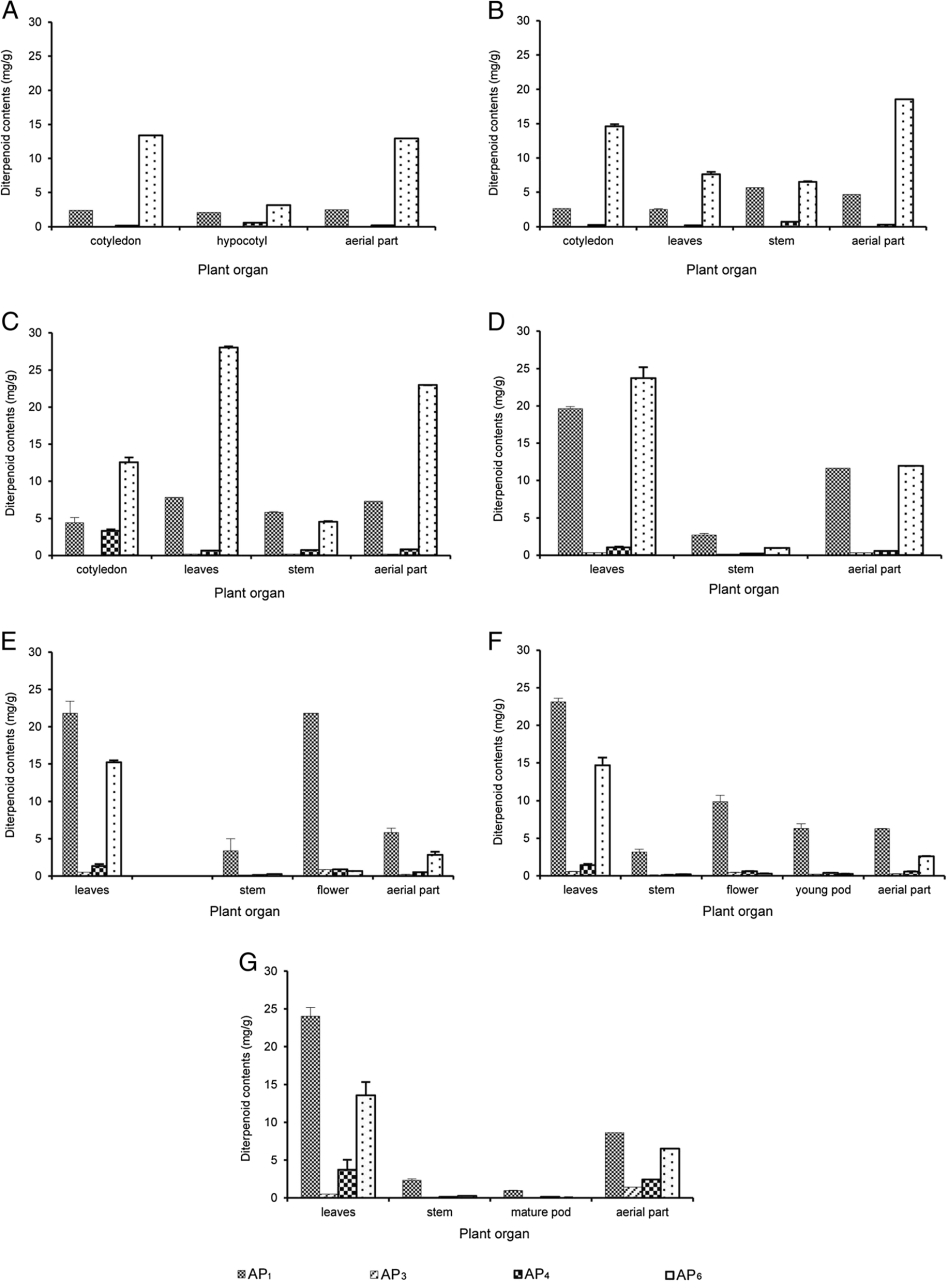
**Contents of four diterpenoids in various tissues of *Andrographis paniculata *at different growth stages (A–G) in greenhouse experiment (September 2006).** Stages were as follows: **A**. Seedling (7 days); **B**. First true leaf (14 days); **C**. Transfer (39 days); **D**. Vegetative (108 days); **E**. 50% Flowering (128 days); **F**. Seed-forming (133 days); and **G**. Mature seed (161 days).

**Figure 4:**
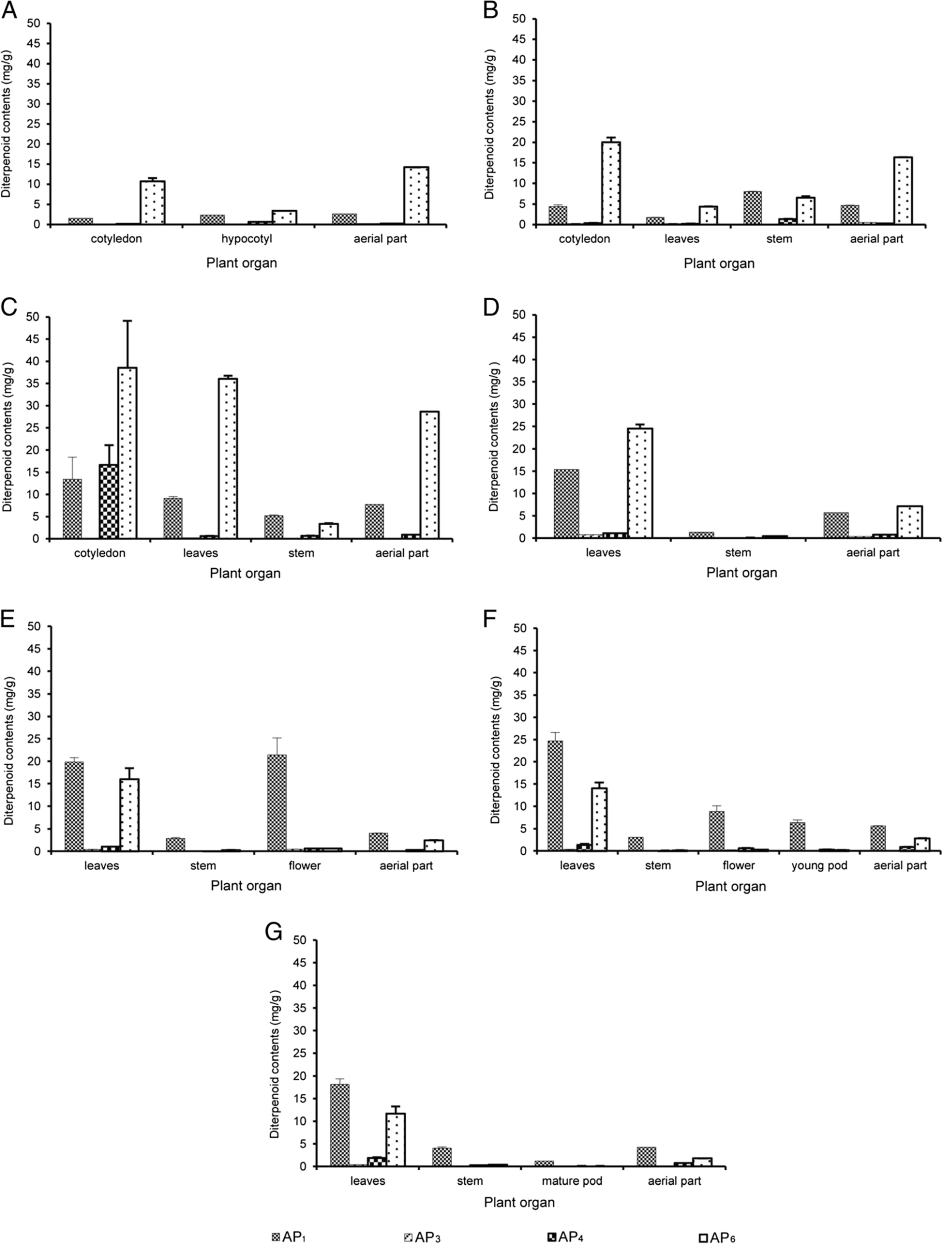
**Contents of four diterpenoids in various tissues of *Andrographis paniculata *at different growth stages (A–G) in greenhouse experiment (January 2007).** Stages were as follows: **A**. Seedling (7 days); **B**. First true leaf (14 days); **C**. Transfer (45 days); **D**. Vegetative (114 days); **E**. 50% Flowering (141 days); **F**. Seed-forming (149 days); and **G**. Mature seed (206 days).

There were large variations in contents of the four active diterpenoids in different plant organs at different growth stages. We studied the patterns of accumulation of these four active diterpenoids in two cultivation periods: September 2006 (Figure 3) and January 2007 (Figure 4). The contents of four active diterpenoids in seeds and roots of *A. paniculata* were lower than limit of detection (LOD). From the seedling to the first true leaf stages, the highest content of AP_6_ was in the cotyledons (Figure 3A and B). At the transfer stage, the highest content of AP_6_ was in leaves both in 2006 and 2007 (28.02 ± 0.15 and 36.05 ± 0.69 mg/g, respectively; Figures 3C and 4C). In 2006, the highest content of AP_1_ was in leaves at the mature-seed stage (24.02 ± 1.18 mg/g) (Figure 3G), whereas in 2007, the highest content of AP_1_ was at the seed-forming stage (24.72 ± 1.89 mg/g) (Figure 4F). Flowers at the 50% flowering stage also contained high contents of AP_1_ in both 2006 and 2007 (21.82 ± 1.61 and 21.42 ± 3.74 mg/g, respectively) (Figures 3E and 4E). In contrast, flowers contained low contents of the other three active diterpenoids (≤ 0.9 mg/g). There were low levels of AP_3_ in all plant organs at all growth stages (≤ 1.4 mg/g). The highest AP_3_ content was at the mature-seed stage in 2006 (1.4 ± 0.12 mg/g) and at the vegetative stage in 2007 (0.77 ± 0.09 mg/g) (Figures 3G and 4D). The highest content of AP_4_ was in cotyledons at the transfer stage in 2006 and 2007 (3.28 ± 0.23 and 16.65 ± 4.48 mg/g, respectively) (Figures 3C and 4C). The range of AP_4_ in leaves was from 0.22 mg/g to 3.72 mg/g and the content increased as plants matured. The highest content of AP_4_ in leaves was at the mature-seed stage in 2006 and 2007 (3.72 ± 1.31 and 1.88 ± 0.15 mg/g, respectively) (Figures 3G and 4G). The AP_6_ content in leaves was lower in 2006 (28.02 ± 0.15 mg/g) than in 2007 (36.05 ± 0.69 mg/g), while the contents of the other three active diterpenoids were similar in both cultivation times. The present results also indicated that the young plants at seedling and first true leaf stages (harvesting time ≤ 14 days) contained high contents of these four active diterpenoids.

### Field experiment

**Figure 5:**
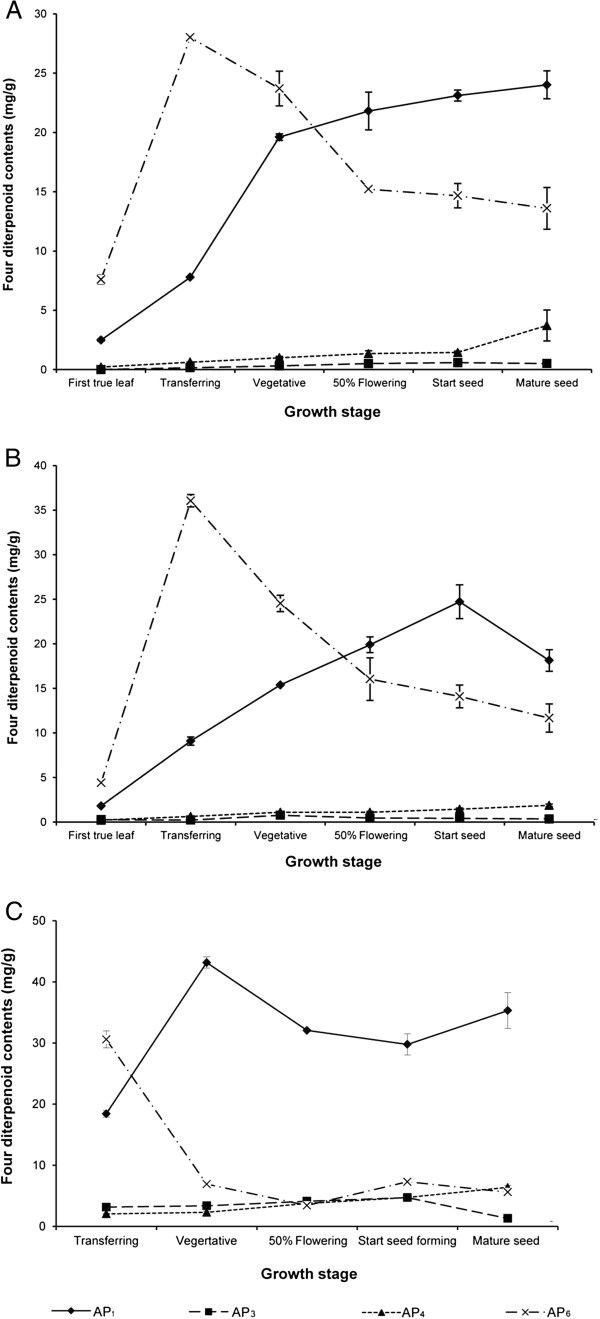
**Contents of four diterpenoids in *Andrographis paniculata *leaves at different growth stages. A**. Greenhouse experiment (September 2006); **B**. Greenhouse experiment (January 2007); **C**. Field experiment.

**Figure 6:**
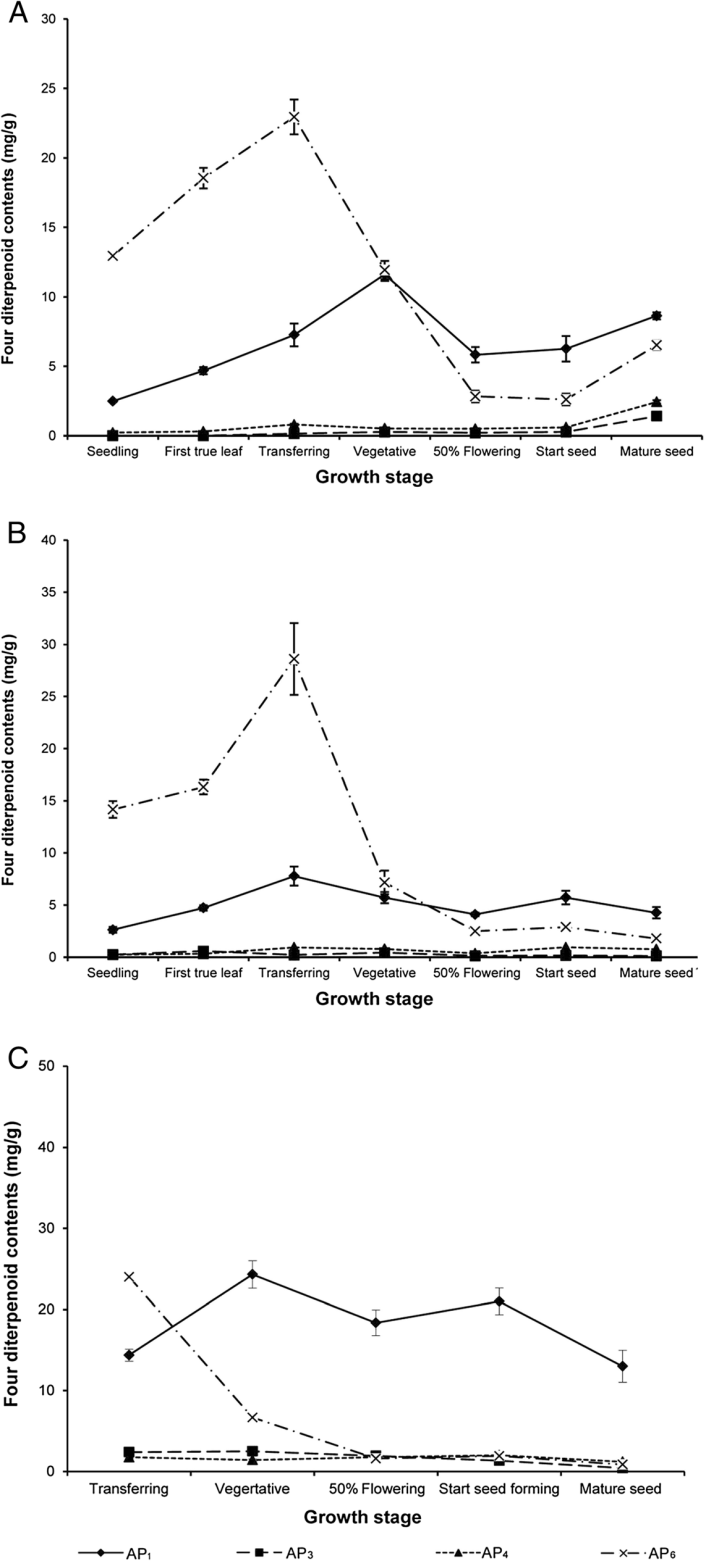
**Contents of four diterpenoids in *Andrographis paniculata *aerial part at different growth stages. A**. Greenhouse experiment (September 2006); **B**. Greenhouse experiment (January 2007); **C**. Field experiment.

In greenhouse and field experiments, the changes in the contents of the four active diterpenoids at different growth stages were similar in leaves and the aerial part (Figures 5 and 6). However, leaves contained higher contents of four active diterpenoids than the aerial part. In the greenhouse experiment, the highest AP_1_ content in leaves (24.02 ± 1.18 mg/g) was about three times higher than that in the aerial part (8.64 ± 0.25 mg/g) at the mature-seed stage in 2006 (Figures 5A and 6A). Similarly, in 2007, the highest AP_1_ content in leaves was four times higher than that in the aerial part at the seed-forming stage (24.72 ± 1.89 mg/g in leaves, 5.71 ± 0.65 mg/g in aerial part) (Figures 5B and 6B). In the field experiment, the highest AP_1_ content in leaves was only twice that in the aerial part at the vegetative stage (43.16 ± 0.92 mg/g in leaves, 24.31 ± 1.68 mg/g in aerial part; Figures 5C and 6C). In greenhouse (2006 and 2007) and field experiments, there was a drastic increase of AP_1_ in leaves from the transfer stage (7.78 ± 0.03, 9.09 ± 0.47, and 18.43 ± 0.54 mg/g, respectively) to the vegetative stage (19.61 ± 0.28, 15.39 ± 0.06, and 43.16 ± 0.92 mg/g, respectively; Figure 5). In the field experiment, the highest content of AP_1_ was at the early growth stage (vegetative stage) whereas it was at the mature-seed and seed-forming stages in the greenhouse experiment in 2006 and 2007, respectively. The highest content of AP_6_ in leaves was at the transfer stage in the field experiment (30.59 ± 1.39 mg/g) (Figure 5C) and in the greenhouse experiment (28.02 ± 0.15 and 36.05 ± 0.69 mg/g in 2006 and 2007, respectively) (Figure 5A, 5B). In the greenhouse experiment, the AP_6_ content in leaves and the aerial part increased markedly from the first true leaf to the transfer stages and then decreased at maturity. The pattern of AP_6_ accumulation in plants was similar in the field and greenhouse experiments (Figures 5 and 6). Moreover, there were low levels of AP_3_ and AP_4_ in leaves and the aerial part at all growth stages in both the greenhouse and field experiments. *A. paniculata* grown in field conditions contained higher levels of the four active diterpenoids than plants grown in greenhouse conditions. For example, AP_1_ contents in leaves at all growth stages (transfer to mature-seed stages) were higher in field-grown plants than in greenhouse-grown plants. Because *A. paniculata* was grown under natural conditions in the field experiment, it is difficult to control cultivation conditions that may affect the levels of these active diterpenoids and quality of this medicinal plant. However, these results showed that the pattern of accumulation of the four active diterpenoids was similar in the field and greenhouse experiments.

To our knowledge, this is the first report to evaluate the contents of these four active diterpenoids in different plant organs during plant development in greenhouse and field experiments. *A. paniculata* is normally harvested at the 50-60% flowering stage, since it was proposed that plants at this stage contain the highest levels of AP_1_[22,23]. Among the active compounds extracted from *A. paniculata*, AP_1_ is generally reported as the major active compound [2]. Matsuda *et al.*[26] analyzed whole *A. paniculata* plants and reported the following diterpenoids contents on a dry weight basis: AP_1_ (0.6%), AP_3_ (0.06%), AP_4_ (0.005%), and AP_6_ (0.02%). Sharma *et al.*[29] reported that the leaves of *A*. *paniculata* contained the highest content of AP_1_ (2.39%), similar to our results (2.4%) in 2006 and 2007 in the greenhouse experiment. However, the highest content of AP_1_ in our field experiment (4.3%) was higher than that reported previously. A new finding in our study was that the most abundant diterpenoid was not AP_1_ but AP_6_ (in 2006 and 2007 at the seedling to vegetative stages in greenhouse experiments, and at transfer stage in the field experiment). The highest content of AP_6_ was found in leaves at the transfer stage; it was four times higher than AP_1_ in 2006 (28.02 ± 0.15 and 7.78 ± 0.03 mg/g, respectively) and 2007 (36.05 ± 0.69 and 9.09 ± 0.47 mg/g, respectively). *A*. *paniculata* at the transfer stage with short harvesting time (39–45 days) contained the highest level of AP_6_, which showed bioactivity as an effective vasorelaxant [16].

Since the four active diterpenoids of *A. paniculata* have different pharmacological properties, knowledge of their different patterns of accumulation among various plant organs and growth stages will be helpful to select plant materials for particular purposes or disease treatments. The patterns of accumulation of AP_1_ and AP_6_ were similar to those reported by Bhan *et al.*[22]. AP_1_ was continuously produced during plant growth while AP_6_ content in leaves peaked at the transfer stage and then decreased as the plants matured. In a previous report, harvesting at 100 days after transplantation was recommended to obtain plants with the highest AP_1_ content [22]. Prathanturarug *et al.*[23] reported levels of AP_1_ and AP_3_ similar to those detected at 50% flowering stage in the present study, although the *A*. *paniculata* plants were harvested at the 60% flowering stage in their study. Recently, Parasher *et al.*[24] analyzed the AP_1_ content in leaves at different growth stages, and found the highest levels of AP_1_ in leaves at 120 days of maturity, similar to our results (highest AP_1_ content was in leaves at mature-seed and seed-forming stages). The content of AP_3_ was lower than that of other diterpenoids in these experiments. However, in our previous study, we observed that the AP_3_ increased in plants and plant products stored for a period of time [25]. Furthermore, the stability of the amorphous form of AP_1_ was temperature-dependent, and it could be converted to AP_3_[30]. Fresh mature plants with low AP_3_ content and high AP_1_ content should be used to treat the common cold, rather than products that have been stored for a period of time, which may exert cardiovascular side effects [31]. The highest content of AP_4_ was at the transfer stage, similar to AP_6_, but in cotyledons rather than leaves. The AP_4_ content in leaves reported in this study was low and slightly increased with maturity. Bhan *et al.*[22] reported that the simultaneous dehydration and glycosylation of AP_1_ formed andrographoside and AP_4_. However, the AP_4_ content in our study was lower than that reported in their study, which may be because of genetic variations and/or cultivation conditions.

## Conclusion

In *A. paniculata*, AP_6_ was at the highest level in leaves at transfer stage in both greenhouse and field experiments. AP_1_ was at the highest level in leaves at vegetative stage and seed-forming stage in field and greenhouse experiments, respectively. The contents of AP_3_ and AP_4_ in leaves were low at all growth stages.

## Abbreviations

AP_1_: Andrographolide; AP_3_: 14-deoxy-11,12-didehydroandrographolide; AP_4_: Neoandrographolide; AP_6_: 14-deoxyandrographolide; HPLC-DAD: High performance liquid chromatography-diode array detector; TLC: Thin-layer chromatography; UV: Ultraviolet; MP: Melting point; IR: Infrared; MS: Mass spectrometer; NMR: Nuclear magnetic resonance; CRI: Chulabhorn Research Institute; %RSD: The percent relative standard deviation; LOD: Limit of detection; LOQ: Limit of quantitation.

## Competing interests

The authors declare that they have no competing interests.

## Authors’ contributions

JS conceived the idea for the study. JS, NR, and NP designed the experiments. NP and NR performed the experiments. NP analyzed the data. JSH cultivated plants in the greenhouse experiment and conducted extractions. SR separated, purified, and identified the four diterpenoids reference standards. NP, NR, and JS wrote the manuscript. All authors read and approved the final version of the manuscript.
